# Pesticide Urinary Metabolite Levels of Children in Eastern North Carolina Farmworker Households

**DOI:** 10.1289/ehp.9975

**Published:** 2007-03-28

**Authors:** Thomas A. Arcury, Joseph G. Grzywacz, Dana B. Barr, Janeth Tapia, Haiying Chen, Sara A. Quandt

**Affiliations:** 1 Department of Family and Community Medicine, Wake Forest University School of Medicine, Winston-Salem, North Carolina, USA; 2 National Center for Environmental Health, Centers for Disease Control and Prevention, Atlanta, Georgia, USA; 3 North Carolina Farmworkers Project, Benson, North Carolina, USA; 4 Department of Biostatistical Sciences, Division of Public Health Sciences and; 5 Department of Epidemiology and Prevention, Division of Public Health Sciences, Wake Forest University School of Medicine, Winston-Salem, North Carolina, USA

**Keywords:** agriculture, biomonitoring, child health, farmworker, health disparities, Latino/Hispanic, occupational health, pesticide exposure, pesticide metabolites

## Abstract

**Background:**

In this investigation we documented the pesticide urinary metabolite levels of farmworker children in North Carolina, determined the number of different metabolites detected for each child, and delineated risk factors associated with the number of metabolites.

**Methods:**

Urine samples were collected from 60 Latino farmworker children 1–6 years of age (34 female, 26 male). Interviews were completed by their mothers in Spanish. We analyzed urine samples for 14 pesticide metabolites, including the organophosphate pesticides chlorpyrifos, coumaphos, diazinon, isazaphos, malathion, pirimiphos, and parathion and its methyl counterpart; a common metabolite of at least 18 pyrethroid insecticides; the repellent DEET; and the herbicides 2,4,5-trichlorphenoxyacetic acid, 2,4-dichlorophenoxyacetic acid, acetochlor, atrazine, and metolachlor. Predictors included measures of paraoccupational, residential, and environmental exposure, child characteristics, and mother characteristics.

**Results:**

Thirteen metabolites were present in the urine samples. Organophosphate pesticide metabolites were detected in a substantial proportion of children, particularly metabolites of parathion/methyl parathion (90.0%; geometric mean 1.00 μg/L), chlorpyrifos/chlorpyrifos methyl (83.3%; geometric mean 1.92 μg/L), and diazinon (55.0%; geometric mean 10.56 μg/L). The number of metabolites detected ranged from 0 to 7, with a mode of 4 detected (28.3%). Boys, children living in rented housing, and children with mothers working part-time had more metabolites detected.

**Conclusions:**

Children in farmworker homes experience multiple sources of pesticide exposure. Pesticides may remain in their environments for long periods. Environmental and occupational health changes are needed to address these exposures. Research is needed with more precise measures of exposure and on the health effects of concurrent exposure to multiple pesticides.

Farmworker children, like their parents, are exposed to pesticides (Arcury et al. 2005, 2006; Coronado et al. 2004; Lambert et al. 2005; Quandt 2004; Strong et al. 2004; Thompson et al. 2003). Pesticide exposure has health consequences for all persons exposed (Reigart and Roberts 1999). Immediate consequences of limited pesticide exposure include rash, nausea, vomiting, and blurry vision. Immediate effects of significant exposure include disorientation, loss of continence, coma, and death. Delayed consequences of limited or significant exposure may include sterility, birth defects, neurodegenerative disease, and cancer (Reigart and Roberts 1999).

The potential consequences of pesticide exposure are greater for children than for adults (Eskenazi et al. 1999; Faustman et al. 2000; Weiss et al. 2004). Because of their short stature and characteristic behaviors, children have greater exposure to pesticides in the environment than do adults. Children have a greater surface to volume ratio than do adults; therefore, they receive a greater dose from the pesticides to which they are exposed. They metabolize toxicants slower than do adults, so the pesticide dose they receive remains with them longer.

The research reporting farmworker children’s exposure to pesticides has considered only the concentrations of dialkylphosphate metabolites of organophosphorus (OP) pesticides (Barr et al. 2004), with one exception (Fenske et al. 2002). Analyses from Washington (Curl et al. 2002; Fenske et al. 2000a, 2000b; Koch et al. 2002; Loewenherz et al. 1997; Thompson et al. 2003), Oregon (Lambert et al. 2005), California (Bradman et al. 2005; Mills and Zahm 2001), the Rio Grande Valley of Texas (Shalat et al. 2003), and North Carolina (Arcury et al. 2005, 2006) indicate that farmworker children are exposed to OP pesticides, and that the concentrations of dialkylphosphate metabolites in their urine are high. However, measurement of the dialkylphosphate metabolites does not provide information on the specific OP pesticides to which these children are exposed (Needham et al. 2005). Further, these analyses do not provide information on the exposure of these children to the non-dialkylphosphate OP pesticides (e.g., acephate) or to other non-OP pesticides. Although knowledge of the general levels of dialkylphosphate OP pesticides in farm-worker children is valuable, knowing specific pesticides to which farmworker children are exposed is important because it will indicate the sources of this exposure. For example, knowing that the metabolite specific to parathion is present in a large percentage of children’s urine samples will direct efforts to identify and then eliminate the sources of parathion exposure.

Only Fenske and colleagues (2002) have reported pesticide-specific metabolite levels in urine samples collected from farmworker children. Focusing on the major metabolites of the OP pesticides chlorpyrifos (TCPy) and parathion (PNP) among 75 children of farm-workers and pesticide applicators, they found TCPy in urine samples from 18 (24%) of the children, and PNP in urine samples from 5 (7%) of the children. PNP had no statistically significant predictors, whereas the only statistically significant predictor of TCPy was living in a household that used an OP pesticide in a garden.

Investigators have proposed a model of farmworker child pesticide exposure that includes paraoccupational, residential, and environmental factors (Fenske et al. 2005; Quandt et al. 2006). Paraoccupational exposure results from contact with persons (e.g., parents) doing farm work. Residential exposure results from contact with agricultural pesticides in the home as well as from the residential application of pesticides. Agricultural pesticides may be brought into the dwelling by workers on clothing, boots, or containers, or directly applied to the dwelling. Characteristics of the dwelling, such as amount of carpeting and general repair, can affect the amount of pesticides that enter and accumulate there. Control of the dwelling and cleaning the dwelling will reduce the amount of agricultural pesticides that accumulate. Environmental exposure results from pesticides that are applied in the larger environment in which the child lives, such as drift during application. Each form of exposure is moderated by safety behaviors exercised by household residents and by characteristics of the child and parents. The implementation of safety behaviors is more likely if parents have received safety training. Safety behaviors include showering immediately after work as well as storage and laundering of soiled work clothes separate from the child’s clothing. Child characteristics, such as sex and age, will modify exposure, because children of different ages and sex have different exposure behaviors. Children of different ages metabolize pesticides differently. Parental characteristics that could modify exposure behaviors include educational attainment and level of employment.

This analysis has two objectives. The first objective is to describe specific urinary pesticide metabolite concentrations for young children living in farmworker households located in eastern North Carolina and the number of metabolites detected for each child. The second objective is to delineate the paraoccupational, residential, and environment risk factors associated with the number of pesticide metabolites present. We used data collected from 60 Latino children, 1–6 years of age, living in eastern North Carolina farm-worker households during the 2004 agricultural production season.

## Materials and Methods

Data for this analysis were collected as part of *Casa y Campo*, a 4-year community-based participatory environmental justice project in which environmental health scientists, health care providers, and farmworker advocates collaborated to reduce pesticide exposure among farmworkers and their families. *Casa y Campo* was implemented in a six-county area of eastern North Carolina, including Duplin, Harnett, Johnston, Sampson, Wake, and Wayne counties. For 2004, the North Carolina Employment Security Commission (Raleigh, NC) estimated that 21,614 migrant and seasonal farmworkers (not counting dependents) worked in these counties during peak harvest, accounting for one-quarter of the 86,040 migrant and seasonal farmworkers in the state. Agriculture in these six counties is diverse (Table 1). Most of the farms in these counties producing tobacco (1,329 farms), sweet potatoes (188 farms), and vegetables (435 farms) would employ migrant and seasonal farmworkers. All of these farms, as well as the large number of farms producing grains, soybeans, and cotton, would use pesticides to which farmworkers and their families could be exposed. Results describing the dialkylphosphate urinary metabolites of OP pesticides for participants in this analysis have been published previously (Arcury et al. 2006).

### Sample and data collection

Sampling, recruitment, and data collection have been described in detail (Arcury et al. 2006). Briefly, from July through August 2004, as part of a larger survey, we recruited 60 households with an adult resident who was employed in farm work and at least one resident child 1–6 years of age. We used a site-based sampling approach to locate and recruit eligible participants (Arcury and Quandt 1999). All 60 eligible households agreed to participate. The mother of each child completed an interview questionnaire, and she was asked to collect a first morning urine void from the resident child between 1 and 6 years of age who was closest to age 5. Respondents were told they would receive a small gift, a bag of food, at the end of the study for completing the interview, and $10 for collecting the urine sample. All participants gave written informed consent. Study procedures were approved by the Wake Forest University School of Medicine and the Centers for Disease Control and Prevention (CDC) Institutional Review Boards.

The interviewer-administered questionnaires were completed in the respondents’ homes in their preferred language, which was Spanish in all cases. Data were collected on respondent and child characteristics, household characteristics, and dwelling quality. Interviews took approximately 25 minutes to complete. At the end of the interview, participants were asked to collect a first morning void from the selected child the next morning. Urine collection materials were left with participants. Each child’s urine sample was picked up from the home by a project staff member the morning the sample was collected, and transported in a cooler with blue ice to our field laboratory, where it was frozen to –20°C. In seven instances, first morning voids were not collected, and the child participant provided a spot void.

### Laboratory analysis

The frozen urine samples were shipped overnight on dry ice to the CDC in Atlanta, Georgia, for analysis. Samples were analyzed using a modification of the method of Olsson et al. (2004). Briefly, 2-mL urine samples were hydrolyzed by enzymes to liberate the glucuronide- or sulfate-bound conjugated metabolites. Hydrolysates were extracted using a mixed-mode solid-phase extraction cartridge. Concentrated extracts were analyzed using high-performance liquid chromatography–tandem mass spectrometry. Two precursor/product ion pairs were analyzed per analyte, one for quantification and one for confirmation. Analyte concentrations were quantified using isotope dilution calibration. Approximately 10% of the samples tested were positive and negative quality control samples.

### Measures

The outcome variables were based on pesticide metabolite concentrations obtained from each child’s urine sample. Metabolites included the OP insecticide metabolites TCPy, 3-chloro-4-methyl-7-hydroxycoumarin (CMHC), 2-isopropyl-4-methyl-6-hydroxypyrimidinol (IMPY), 5-chloro-1,2-dihydro-1-isopropyl-[3H]-1,2,4-triazol-3-one (CIT), malathion dicarboxylic acid (MDA), PNP, and 2-diethylamino-6-methyl pyrimidin-4-ol (DEAMPY), the pyrethroid insecticide metabolite 3-phenoxy-benzoic acid (3PBA), diethyl-m-toluamide (DEET) repellent, and the herbicide metabolites 2,4,5-trichlorophenoxyacetic acid (2,4,5-T), 2,4-dichlorophenoxyacetic acid (2,4-D), acetochlor mercapturate (ACE), atrazine mercapturate (ATZ), and metolachlor mercapturate (MET). Measures included the concentrations of each metabolite and the total number of different pesticide metabolites detected in each child’s urine sample.

We constructed measures for each domain in the model of pesticide exposure. Measures of paraoccupational exposure included the following variables: whether mother was currently employed doing farm work, whether father was currently employed doing farm work, and number of farmworkers in the household (1, 2, ≥ 3). Residential exposure measures included home ownership (rent, own, other), ease of cleaning (easy, difficult), number of bathrooms (1, 2, 3), and number of rooms with carpet (0, 1–2, ≥ 3). Proximity to nearest agricultural field was the environmental exposure variable; it had the values of adjacent when agricultural fields directly abutted or were across the road from the property on which the respondent’s dwelling was located, and nonadjacent when agricultural fields did not abut the property on which the respondent’s dwelling was located.

Safety behavior measures included the following: parental pesticide safety training (mother and father each coded no farm work, farm work and training, farm work and no training), whether any farmworker in the household regularly delayed showering after work for > 15 min, whether any farmworker in the household regularly changed work clothes inside the dwelling, storage of soiled farm work clothes (everyone stores clothes outside, anyone stores clothes inside, anyone stores work clothes with other clothes), and whether everyone launders soiled farm clothes separately. Child and parental characteristics included child sex and age (1 or 2 years, 3 or 4 years, 5 or 6 years), mother’s education (≤ 6 years, 7 to 9 years, ≥ 10 years), and mother’s current employment (none, part time, full time).

### Data analysis

Medians and geometric means (when the number of detects was > 50%), unadjusted and adjusted for creatinine, were calculated for the individual 14 urinary pesticide metabolites. Concentrations below the analytic limits of detection (LOD) were substituted by the LOD divided by the square root of 2 (Homung and Reed 1990). To identify potential predictors for the number of detects, we examined bivariate associations between the outcome and the predictor variables through analysis of variance (ANOVA). Only the variables whose *p*-values were < 0.20 were considered in further model selection process. Finally, a model with three variables (mother’s employment, child’s sex, and ownership) was prescribed by a forward selection procedure. Analyses were repeated with creatinine-adjusted measures to control for the seven children who gave spot voids. The results did not differ, and the results for non-creatinine–adjusted measures are presented. All analyses were performed using SAS version 9.0 (SAS Institute Inc., Cary, NC).

## Results

### Exposure, safety behavior, and personal characteristics

The 60 children considered in this analysis varied in paraoccupational, residential, and environmental sources of pesticide exposure, as well as safety behaviors and personal characteristics (Table 2). Most of their mothers (68.3%) and fathers (56.7%) were employed as farmworkers at the time of data collection. More than two-thirds (36.7%) lived in households with two farmworkers, and 25.0% lived in households with three or more farmworkers. Most (53.3%) of the children lived in rented homes, whereas 33.4% lived in farmworker-owned homes and 13.3% lived in homes of other tenure. More than half (58.3%) lived in homes that their mothers described as easy to clean, and more than half (53.3%) of these children lived in dwellings with one bathroom. Most (91.7%) lived in dwellings with at least one carpeted room, and almost 70% lived in dwellings with three or more carpeted rooms. The dwellings in which over half (56.7%) of the children lived were not adjacent to agricultural fields.

Twenty-three of 41 mothers (38.3% of total sample) and 17 of the 34 fathers (28.3% of total sample) employed in farm work had not received pesticide safety training. Three-fifths of the children lived in households in which individuals employed in farm work did not shower immediately after work, and 83.3% lived in households in which individuals changed out of their soiled farm work clothes inside the dwelling. However, 15% lived in households in which farm work clothes were stored with other laundry, and 20% lived in households in which farm work clothes were laundered with other work clothes.

More of these children were girls (56.7%) than boys (43.3%). Most were 3 or 4 years of age (60.0%), with one-third 5 or 6 years of age, and 8.3% 1 or 2 years of age. More than half of their mothers had less than a secondary education. About one-quarter of their mothers did not work outside the home, with one-quarter working part-time and one-half working full-time.

### Pesticide metabolite levels

The metabolites for 13 of the 14 pesticides were present in the urine samples of the 60 children (Table 3). Among these metabolites were those of seven OP insecticides. PNP was the most common OP insecticide present; PNP was present in 90% of the samples, and had a geometric mean (GM) of 1.0 μg/L unadjusted for creatinine, and 1.25 μg/g adjusted for creatinine. TCPy was present in 83.3% of the samples, IMPY was present in 55.0% of the samples, and MDA was present in 28.3% of the samples. The GM for TCPy was 1.92 μg/L unadjusted for creatinine, and 2.38 μg/g adjusted for creatinine; and the GM for IMPY was 0.56 μg/L unadjusted for creatinine, and 0.70 μg/g adjusted for creatinine. The median for MDA was 0.21 μg/L unadjusted for creatinine, and 0.33 μg/g adjusted for creatinine. CMHC was detected in seven (11.7%) of the samples, DEAMPY in three (5.0%) of the samples, and CIT in one (1.7%) of the samples.

The pyrethroid insecticide metabolite 3PBA was present in 40.0% of the farm-worker child urine samples. The metabolite for DEET repellent was present in 10.0% of the farmworker child urine samples. The herbicide 2,4,5-T was present in one (1.7%) of the samples. The herbicide 2,4-D was present in 41.7% of the samples, ACE was present in 21.7% of the samples, and ATZ was present in 6.7% of the samples.

### Pesticides detected per child: number and predictors

The number of metabolites detected in the children’s urine samples varied from zero to seven. One child (1.7%) had no detects, five children (8.3%) had one detect, one child (1.7%) had two detects, 16 children (26.7%) had three detects, 17 children (28.3%) had four detects, eight children (13.3%) had five detects, nine children (15.0%) had six detects, and three children (5.0%) had seven detects.

Three of the predictors had statistically significant associations with the mean number of pesticides detected in the bivariate analysis (Table 4). Children residing in rented homes had 4.41 pesticides detected in their urine samples, compared with 3.65 detects among those living in an owned home and 3.00 detects among those living in other homes. Boys had 4.46 pesticides detects, compared with 3.59 for girls. Finally, children of mothers working part-time had 5.06 detects, compared with 3.63 detects among children whose mothers worked full-time and 3.43 detects among children whose mothers did not work. In the multivariate analysis, mother’s employment remained significantly associated with number of detects, whereas the association of child sex trended toward significance and home ownership was not significant.

## Discussion

Urine samples from most study children living in farmworker homes have a variety of pesticide metabolites. At least one specific pesticide metabolite was found in the urine samples for 59 of these 60 children; the urine samples for 88.3% of these children had three or more specific pesticide metabolites present. Analysis of the general dialkylphosphate OP pesticide metabolites for these same children found at least one of these six metabolites in every sample (Arcury et al. 2006).

The specific pesticide metabolites present indicate multiple sources and pathways of potential exposure among children in farm-worker homes, and illustrate the length of time these pesticides remain in the environments of farmworker children. The pesticide metabolites present in these children’s urine indicate at least four pathways of exposure: *a*) a paraoccupational take-home pathway in which workers bring pesticides into their homes on their person or on their clothing; *b*) an environmental pathway in which pesticides applied to nearby fields drift into the residential environment; *c*) a residential pathway of pesticides applied in the home; and *d*) a residual pathway in which pesticides deposited inside the home from any of the pathways at an earlier time remain active. Earlier research in North Carolina showed that farmworkers had little knowledge of the pesticides used where they work, and that they did not have information about pesticides applied to dwellings that were rented or grower-provided (Arcury et al. 2001; Early et al. 2006). Drift has been shown to result in the dispersal of agricultural pesticides to dwellings on surrounding land (Fenske et al. 2000b; Ward et al. 2006; Weppner et al. 2006). Finally, agricultural communities have generally higher levels of environmental pesticides compared with nonagricultural communities, with residues from discontinued pesticides found in contemporary rural environments and homes (Lu et al. 2004; Quandt et al. 2004; Wolz et al. 2003). However, the possible sources and pathways of exposure for several pesticide metabolites found in the samples from these children remain unclear.

In discussing the sources and pathways of potential exposure, it is important to remember that the urine samples used in this analysis were collected in June and July 2004. Exposure of these children to pesticides in Mexico should be discounted as the source of the metabolites; these are the children of seasonal rather than migrant farmworkers, who have been settled in North Carolina for several years. Many of these children were born in the United States, and have limited direct or indirect contact with sources of exposure from Mexico.

Chlorpyrifos and diazinon have been used for residential as well as agricultural applications. However, chlorpyrifos was banned for residential use by the end of 2001, and diazinon was banned for residential use at the end of 2004. Fifty of the 60 children had TCPy, the chlorpyrifos metabolite, in their urine. This would indicate that chlorpyrifos exposure for these children did not result from recent residential application, but could result from the take-home and drift pathways as well as from residual deposition. The diazinon metabolite IMPY was present in 33 of the 60 samples. Because it was still available for residential use at the time these data were collected, it is possible that diazinon exposure could result from any of the pathways.

Malathion has outdoor residential use, whereas parathion has no residential use. Yet 54 of the 60 children had the parathion metabolite PNP and 17 had the malathion metabolite MDA. Farmworkers may be exposed to malathion at work (e.g., picking peppers), but it is unlikely that they would work in fields to which parathion has been applied (e.g., cotton). Therefore, exposure resulting in the presence of PNP is most likely the result of the drift and residual deposition pathways, whereas MDA could result from the take-home, drift, or residual pathways.

Pyrethroid insecticides are widely used for residential and agricultural applications. The 24 children with 3PBA, the pyrethorid metabolite, in their urine sample could have been exposed through any of the pathways. The herbicides 2,4-D and acetochor are widely used for residential and agricultural applications, and their metabolites could result from exposure through any of the pathways.

Atrazine is used on corn (85% of all use), sorghum (10%), and sugar cane (3%). Three-quarters of all corn has atrazine applied. Corn and sorghum are grown in North Carolina. However, field corn and sorghum are machine cultivated and harvested. Sweet corn is hand picked, but few farmworkers are employed for this task. The most plausible explanation for four farmworker children having the metabolite for atrazine in their urine can be limited to drift or contaminated water supplies.

The sources and pathways of exposure among farmworker children to the OP pesticides coumaphas, pirimiphos methyl, and isazophos, and to the herbicide 2,4,5-T are not apparent. Coumaphas is used to control insects on livestock, and farmworkers in North Carolina seldom work with livestock. However, CMHC, the metabolite of coumaphas, was detected in samples from seven of the children. Pirimiphos methyl is used postharvest to treat stored corn and sorghum grain, activities with limited farmworker participation. Yet DEAMPY, the metabolite of pirimiphos methyl, was detected in samples from three children. Isazophos was used to treat lawns and turf; use of isazophos was cancelled in North Carolina at the end of 1998, and cancelled by the U.S. Environmental Protection Agency (EPA) in mid-1999. Yet CIT, the metabolite of isazophos, was found in one child. Finally, the herbicide 2,4,5-T has been banned for use in the United States since 1986, years before most Latino farm-workers had immigrated to North Carolina. The metabolite for 2,4,5-T was detected in the sample of one child. The metabolites for isazophos and 2,4,5-T were found in samples from different children.

Few data exist with which our results can be compared. The 1999–2000 National Health and Nutrition Examination Survey (NHANES) data provide results for the proportion of detects and levels detected for the OP pesticide metabolites TCPy, IMPY, MDA, and PNP (Barr et al. 2005). Caution must be taken in the comparison of our results with those reported for the NHANES sample. Development is a major factor affecting pesticide metabolism; and our participants were 1–6 years of age, whereas the youngest age group included in the NHANES sample was 6–11 years of age (Table 5). For TCPy, the percent of children with pesticide metabolite detected in the entire 1999–2000 NHANES sample (91%), among the Mexican-American sample (87%), and among children 6 to 11 years of age (97%) was somewhat greater than for the children in our sample (83%), with the GMs, 50th percentile, and 95th percentile (creatinine unadjusted and adjusted) being lower for the entire and Mexican-American samples, but higher among children 6 to 11 years of age. The percent detects for IMPY and PNP was higher for our sample (55% and 90%, respectively) compared with the NHANES samples (total sample, 29%, 22%; Mexican-American sample, 24%, 34%; children 6 to 11 years of age sample, 26%, 26%). The 95th percentile of IMPY for our sample (3.94 μg/L unadjusted for creatinine, 3.01 μg/g adjusted for creatinine) approximates those for the NHANES samples. The 95th percentile for PNP is greater among the children in our sample (6.32 μg/L unadjusted for creatinine, 7.99 μg/g adjusted for creatinine) than for the entire NHANES sample (5.0, 4.2) and among children 6 to 11 years of age (4.2, 4.2). However, the 95th percentile is much higher in the NHANES Mexican-American sample (21, 17). The percent detects for MDA for the children in our sample (28%) is lower than the percent detects in the entire 1999–2000 NHANES sample (52%), among the Mexican-American sample (61%), and among children 6 to 11 years of age (56%). However, the 95th percentile (6.87 μg/L unadjusted for creatinine, 8.64 μg/g adjusted for creatinine) for the children in our sample is greater than that for the entire NHANES sample (1.6, 1.8), the NHANES Mexican-American sample (1.6, 1.7), and among children 6 to 11 years of age (2.8, 3.7). No comparison data are available for the pyrethroid insecticide metabolite 3PBA, DEET repellent, or any of the herbicide metabolites.

Fenske and colleagues (2002) reported on the presence and levels of two OP pesticide metabolites for children residing in Washington State agricultural households. Not all of the children lived in farmworker households. They found TCPy in urine samples from 24% (18 of 75) children, with level of 6.0 μg/L (SD = 17) among children residing within 200 feet of an agricultural field and 1.3 μg/L (SD = 4.9) for those living more than 200 feet from a field. This metabolite was present in 83.3% of our samples, and had a GM of 1.92 μg/L. They reported PNP in urine samples for 7% (5 of 75) of the children. The comparable mean PNP levels were 33 μg/L (SD = 210) and 0. This metabolite was present in 90.0% of our samples, and had a GM of 1.00 μg/L. These differences reflect regional differences in agricultural uses.

Earlier studies have not considered the number of different pesticide metabolites present in individual children. Combining doses from several pesticides in a single child may result in interactions beyond the effects of a single toxicant (Eskenazi et al. 1999). Among these 60 children, 54 had two or more pesticide metabolites, with nine children having six pesticide metabolites and three children having seven pesticide metabolites. There was no discernable pattern in the combinations of pesticide metabolites present in the samples. Boys versus girls, and those living in rented housing versus owned or other housing had a greater number of pesticide metabolites in bivariate analysis. This may reflect sex differences in behavior. More important, it reflects how control of a dwelling may decrease exposure. Children whose mothers worked part-time versus full-time or who did not work had a greater number of pesticide metabolites in the bivariate and multivariate analysis. The interpretation of this association is not clear. We conducted further analysis to examine whether mothers working part-time were more likely to be employed doing farm work; this was not the case. It is possible that mothers working part-time are less able then unemployed women to attend to household hygiene, whereas mothers working full-time could count on more help in home care. Research is needed that has greater precision in measuring of pesticide exposure risk factors (Arcury et al. 2006; Quandt et al. 2006).

The results of this study should be considered in light of its limitations. The cross-sectional design limits analysis to association rather than causation. The lack of environmental pesticide samples does not allow for consideration of the location of exposure. Data on residential pesticide application were not collected. The metabolites present in the urine may also include exposure to the less toxic environmental degradates of the target pesticides. The determination of whether a metabolite was detected in a child’s urine sample, and therefore the total number of metabolites detected in a child, may be limited by the LOD of current analytic procedures; therefore, the number of metabolites detected is a conservative estimate. Finally, the measurement of risk factors may lack precision. This study also has several strengths. It is based on a relatively large sample of children. It is one of the few studies of farmworker child exposure on the East Coast. Finally, it is one of only a few studies to examine a large number of specific pesticide metabolites for farmworker children.

Several implications for environmental and occupational health practice and research can be drawn from our results. Pesticide exposure pathways for farmworkers and their children are multiple and complex. Pathways include take-home, drift, residential application, and residues. The sources of exposure to several of the pesticide metabolites found in the urine samples are not clear. Farmworkers must be educated about the take-home pathway. Current U.S. EPA Worker Protection Standard training (U.S. EPA 1992), when workers receive it (Arcury et al. 2001; U.S. Government Accountability Office 2000), provides little information about pesticides that can be taken home on the clothes, boots, and skin of workers, and about how to reduce or eliminate the amount taken home. Regulations on pesticide application to reduce drift must be reviewed. Farmworker and all rural families must be educated about drift and how to reduce exposure. Farmworker housing regulations must be improved to reduce the need for residential pesticide application (Early et al. 2006; Housing Assistance Council 2001). Farmworkers need to be educated about alternatives to using pesticides, such as residential integrated pest management. Farmworker and other rural homes need to be deep cleaned to remove pesticides from indoor environments. Procedures for this deep cleaning need to be developed and tested (McCauley et al. 2006a).

Research is needed that includes more precise measures of exposure pathways. Laboratory techniques measuring pesticide metabolites in urine and other matrices as well as environmental monitoring have improved substantially (Barr et al. 2006; Hoppin et al. 2006). However, measurement of exposure pathways in epidemiologic research has not improved greatly. For example, questions about recent employment as a farmworker must be changed to questions about the amount of farm work conducted in the 3 days before samples for biomonitoring are collected.

Research on the health effects of concurrent exposure to multiple individual pesticides and classes of pesticides is also needed. Current knowledge of health effects of pesticides in general is limited, but documentation is beginning (McCauley et al. 2006b). Examining health effects in light of the interactions of several different pesticides will provide greater insight into the actual risks to those exposed.

## Figures and Tables

**Table 1:** Selected agricultural characteristics of counties from which participants were recruited, 2002 Census of Agriculture.^a^

			No. of farms producing						
County	No. of farms	Acres in farmland	Tobacco	Sweet potatoes	Vegetables	Corn for grain	Wheat for grain	Soybeans	Cotton
Duplin	1,190	234,658	213	9	74	341	137	401	58
Harnett	740	114,361	144	19	55	157	44	173	38
Johnston	1,144	194,211	360	91	76	152	108	431	56
Sampson	1,178	298,483	243	44	135	279	162	431	126
Wake	846	92,803	161	18	51	22	54	102	1
Wayne	722	171,449	208	7	44	187	134	296	80

a Data from U.S. Department of Agriculture (2007).

**Table 2:** Exposure characteristics of children 1 to 6 years of age living in farmworker families, eastern North Carolina, 2004.

Exposure characteristics	No. (%)
Paraoccupational exposure	
Mother does farm work	41 (68.3)
Father does farm work	34 (56.7)
No. of farmworkers in house	
1	23 (38.3)
2	22 (36.7)
3 or more	15 (25.0)
Residential exposure	
Home ownership	
Own	20 (33.3)
Rent	32 (53.3)
Other	8 (13.3)
Ease of cleaning	
Easy	35 (58.3)
Difficult	25 (41.7)
No. of bathrooms	
1	32 (53.3)
2 or 3	28 (46.7)
No. of rooms with carpet	
0	5 (8.3)
1 or 2	14 (23.4)
3 or more	41 (68.3)
Environmental exposure: proximity to nearest agricultural field	
Adjacent	26 (43.3)
Nonadjacent	34 (56.7)
Safety behaviors	
Mother’s pesticide safety training	
No farm work	19 (31.7)
Farm work and training	18 (30.0)
Farm work and no training	23 (38.3)
Father’s pesticide safety training	
No farm work	26 (43.3)
Farm work and training	11 (18.3)
Farm work and no training	17 (28.3)
Anyone in house delays shower after farm work	36 (60.0)
Anyone in house changes farm clothes inside	50 (83.3)
Farm work clothes storage	
Everyone stores clothes outside	11 (18.3)
Anyone stores clothes inside	40 (66.7)
Anyone stores clothes with other clothes	9 (15.0)
Everyone launders farm clothes separately	48 (80.0)
Child and mother characteristics	
Child sex	
Female	34 (56.7)
Male	26 (43.3)
Child age (years)	
1 or 2	5 (8.3)
3 or 4	36 (60.0)
5 or 6	19 (31.7)
Mother’s education (years)	
1 to 6	34 (56.7)
7 to 9	15 (25.0)
≥ 10	11 (18.3)
Mother’s employment	
None	14 (23.3)
Part-time	16 (26.7)
Full-time	30 (50.0)

**Table 3:** Pesticide urinary metabolites for children 1–6 years of age (*n* = 60) in eastern North Carolina farm-worker households, 2004.

			Creatine unadjusted (μg/L)		Creatinine adjusted (μg/g creatinine)	
Pesticide urinary metabolites	LOD (ng/mL)	Detects no. (%)	Median	GM	Median	GM
Organophosphorus insecticides						
TCPy (chlorpyrifos)	0.2	50 (83.3)	2.47	1.92	3.38	2.38
CMHC (coumaphos)	0.2	7 (11.7)	0.14	—	0.17	—
IMPY (diazinon )	0.7	33 (55.0)	0.49	0.56	0.60	0.70
CIT (isazophos)	1.5	1 (1.7)	1.06	—	1.19	—
MDA (Malathion)	0.3	17 (28.3)	0.21	—	0.33	—
PNP (parathion)	0.1	54 (90.0)	1.55	1.00	1.53	1.25
DEAMPY (pirimiphos methyl)	0.2	3 (5.0)	0.14	—	0.17	—
Pyrethroid insecticides						
3PBA	0.1	24 (40.0)	0.07	—	0.15	—
DEET repellent	0.1	6 (10.0)	0.07	—	0.08	—
Herbicides						
2,4,5-T	0.1	1 (1.7)	0.07	—	0.08	—
2,4-D	0.2	25 (41.7)	0.14	—	0.23	—
ACE (acetochlor)	0.1	13 (21.7)	0.07	—	0.09	—
ATZ (atrazine)	0.3	4 (6.7)	0.09	—	0.10	—
MET (metolachlor)	0.2	0 (0.0)	ND	ND	ND	ND

Abbreviations: —, the small number of detects (< 50%) make the GM an unreliable measure for this metabolite; ND, no detects.

**Table 4:** Predictors with a significant relationship to number of pesticide urinary metabolite detects, children 1 to 6 years of age (*n* = 60) in eastern North Carolina farmworker households, 2004.

		Significance	
Characteristic	No. of detects (mean ± SE)	Bivariate analysis^a^	Multivariate analysis^a^
Home ownership		0.045	[Table-fn tfn3-ehp0115-001254]—
Own	3.65 ± 0.35		
Rent	4.41 ± 0.27		
Other	3.00 ± 0.54		
Child sex		0.035	0.078
Male	4.46 ± 0.31		
Female	3.59 ± 0.27		
Mother’s employment		0.004	0.008
None	3.43 ± 0.39		
Part-time	5.06 ± 0.37		
Full-time	3.63 ± 0.27		

—, not significant.

a Other variables considered in the bivariate analysis and included in the multivariate analysis were mother does farm work, father does farm work, number of farmworkers in house, ease of cleaning house, number of bathrooms, number of rooms with carpet, proximity to nearest agricultural field, mother’s pesticide safety training, father’s pesticide safety training, anyone in house delays shower after farm work, anyone in house changes farm clothes inside, farm work clothes storage, everyone launders farm clothes separately, child age, and mother’s education.

**Table 5:** Comparison of percent detects, creatinine unadjusted and adjusted geometric mean, 50th percentile, and 95th percentile, for children 1–6 years of age (*n* = 60) in eastern North Carolina farmworker households, 2004, and 1999–2000 NHANES*a* data for total, Mexican-American, and children 6–11 years of age.

	Creatinine unadjusted				Creatinine adjusted		
Metabolites and samples	Percent detects	GM	50th	95th	GM	50th	95th
TCPy							
Eastern North Carolina farmworker children 1999–2000 NHANES*a*	83.3	1.92	2.47	16.91	2.38	3.4	17.46
Total	91	1.77	1.7	9.9	1.58	1.47	8.4
Mexican Americans	87	1.61	1.67	7.4	1.46	1.44	5.8
Children 6–11	97	2.88	2.7	16	3.11	3.2	14
IMPY							
Eastern North Carolina farmworker children 1999–2000 NHANES*a*	55.0	0.56	0.49	3.94	0.70	0.60	3.01
Total	29	—	—	3.7	—	—	3.4
Mexican Americans	24	—	—	4.2	—	—	3.9
Children 6–11	26	—	—	3.6	—	—	5.1
MDA							
Eastern North Carolina farmworker children 1999–2000 NHANES*a*	28.3	—	0.21	6.87	0.57	0.34	8.64
Total	52	—	< LOD	1.6	—	< LOD	1.8
Mexican Americans	61	—	< LOD	1.6	—	< LOD	1.7
Children 6–11	56	—	0.49	2.8	—	0.44	3.7
PNP							
Eastern North Carolina farmworker children 1999–2000 NHANES*a*	90.0	1.00	1.55	6.32	1.25	1.52	7.99
Total	22	—	—	5	—	—	4.2
Mexican Americans	34	—	—	21	—	—	17
Children 6–11	26	—	—	4.2	—	—	4.2

a Data from Barr et al. 2005.
